# Extended versus limited pelvic lymph node dissection and long‐term outcomes in high‐risk prostate cancer before radiotherapy

**DOI:** 10.1002/bco2.70193

**Published:** 2026-03-22

**Authors:** Georgios Daouacher, Jessica Carlsson, Pernilla Sundqvist, Mauritz Waldén

**Affiliations:** ^1^ Faculty of Medicine and Health Örebro University Örebro Sweden; ^2^ Department of Urology Central Hospital of Karlstad Karlstad Sweden; ^3^ Department of Urology, Faculty of Medicine and Health Örebro University Örebro Sweden

**Keywords:** external beam radiation therapy, high‐risk prostate cancer, pelvic lymph node dissection, prostate cancer: biochemical recurrence

## Abstract

**Objectives:**

To compare oncological outcomes after extended pelvic lymph node dissection (PLND) versus limited in patients with high‐risk prostate cancer (PCa) undergoing curative external beam radiation therapy (EBRT).

**Patients and Methods:**

From 3627 men with PCa at a single centre between 2000 and 2013, 167 with high‐risk, age ≤75, Gleason score 6–10, clinical stage T1–T3, PSA < 100 ng/ml, no distant metastases (M1) and node‐negative at the obturator fossa, underwent PLND before curative EBRT. Of these, 90 received limited, and 77 underwent extended PLND. Mean follow‐up (SD) was 14.9 yr (5.8) for the limited and 12.3 yr (3.3) for the extended PLND. Primary endpoint was biochemical recurrence (BCR), secondary M1, cancer–specific mortality (CSM), overall mortality (OM). HR, KM and Cox regression models adjusted for age and Cambridge prognostic group (CPG) score. RR, RD at 11 yr.

**Results:**

Extended PLND was associated with a significantly lower risk for BCR (HR: 0.51, 95% CI: 0.31–0.86, *p =* 0.01) (RR: 0.43, 95% CI: 0.26–0.69, *p = 0.001*), lower risk of M1 (HR: 0.22, 95% CI: 0.08–02.65, *p = 0.006*) (RR: 0.26, 95% CI: 0.09–0.73, *p = 0.004*) and lower CSM compared with limited PLND (HR: 0.31, 95% CI: 0.08–0.65 *p* = 0.035) (RR 0.27, 95% CI: 0.08–0.91, *p* = 0.028). OM did not differ significantly.

**Conclusions:**

Extended PLND prior to curative ERBT shows reductions in BCR, M1 and CSM long‐term outcomes following extended versus limited PLND. Extended PLND can be considered in cases with high‐risk PCa prior to curative EBRT.

## 1 INTRODUCTION

Pelvic lymph node dissection (PLND) remains the most reliable method for assessing nodal involvement and determining prognosis in prostate cancer (PCa).^1^, ^2^, ^3^, ^4^ Consequently, PLND is frequently incorporated into surgical treatment strategies during radical prostatectomy. However, it remains uncertain whether extended PLND, defined by the removal of a greater number of lymph nodes,^5^ provides a survival benefit. This potential benefit could stem from the clearance of overt lymph node metastases^6^ or the removal of occult micro metastatic disease.^7^

Two recent randomized trials (RTs) have failed to demonstrate significant differences in survival outcomes between patients undergoing limited versus extended PLND at time of radical prostatectomy.^8^, ^9^ However, a 2024 update of the trial by Touijer et al. reported improved metastasis‐free survival in patients treated with extended PLND.^10^ These findings are consistent with those of a recent retrospective analysis involving 2346 patients, which compared radical prostatectomy with extended PLND to radical prostatectomy alone. The study showed a significant improvement in metastasis‐free survival in favour of extended PLND, particularly among patients with high‐risk PCa.^11^

However, these studies are limited to patients undergoing radical prostatectomy, and the role of extended PLND in the setting of radiotherapy remains uncertain.^12^ It is plausible that performing extended PLND prior to external beam radiation therapy (EBRT) with curative intent could reduce disease burden and improve survival outcomes. This distinction is clinically relevant, as the mechanisms underlying local disease control and immune modulation differ between surgical^13^ and radiotherapeutic approaches.^14^ Nevertheless, evidence supporting this strategy is scarce, as PLND prior to EBRT is no longer considered the standard of care.^15^

Until 2008, hormonal therapy alone was the standard of care for patients with high‐risk PCa. The SPCG‐7 subsequently demonstrated a survival benefit from adding prostate‐directed EBRT to hormonal treatment.^16^ Following this, EBRT was typically reserved for patients with node‐negative (N0) status, as determined by PLND limited to the obturator fossa, yielding only a small number of lymph nodes. In 2008, Mattei et al. proposed a standardized template for extended PLND, involving a broader anatomical dissection range.^2^ This development prompted renewed interest in whether the additional removal of lymph nodes through extended PLND could offer clinical benefit. Extended PLND became the new standard in 2008, as described by Mattei.^2^ After the adoption of this new surgical technique, patients continued to be eligible for EBRT with curative intent if no lymphatic spread was proven in the obturator fossa (limited PLND), regardless of the nodal status provided by extended PLND.

The aim of this study was to evaluate long‐term outcomes following limited versus extended PLND in patients with high‐risk PCa and N0 status in the obturator fossa, prior to curatively intended EBRT. The primary endpoint was biochemical recurrence (BCR), while secondary endpoints included the occurrence of distant metastasis (M1), cancer‐specific mortality (CSM) and overall mortality (OM).

## 2 PATIENTS AND METHODS

### 2.1 Study design

This is a retrospective, observational, single‐centre study, evaluating outcomes of limited versus extended PLND performed prior to curatively intended ERBT in patients with high‐risk PCa. Data were collected from the hospital's patient records and the national prostate cancer registry. Details regarding EBRT were sourced from the hospital's treatment database. Patients were followed up every six months during the first 2 yr, and annually thereafter.

The study was approved by the Regional Ethics Committee in Uppsala, Sweden (EPN 2015‐410B), which determined that patient consent was not required.

### 2.2 Patients

**FIGURE 1:**
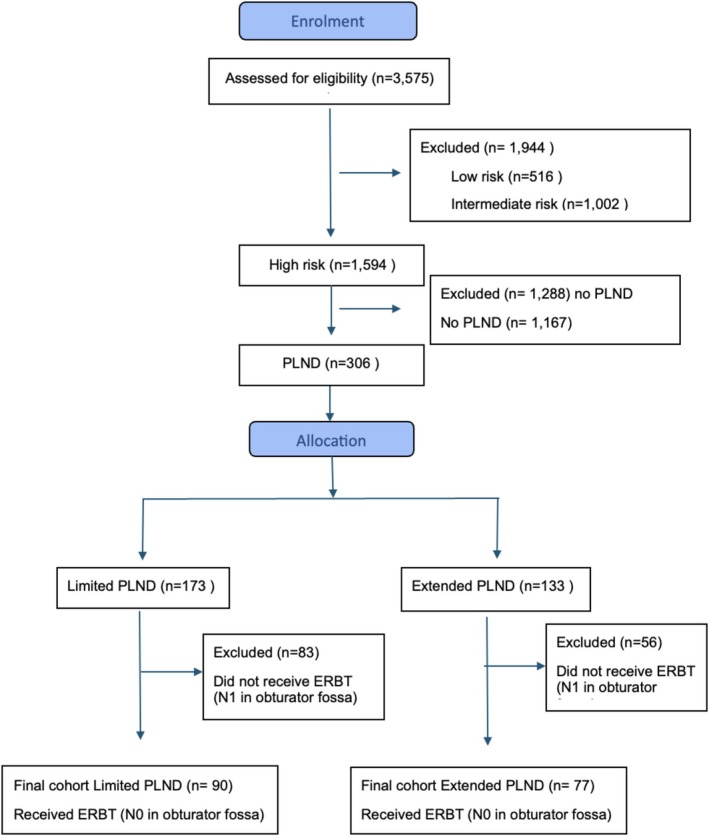
Flow diagram of patient enrolment.

Among 3627 newly diagnosed PCa cases at a single tertiary centre (2000–2013), 90 men underwent limited PLND, and 77 men underwent extended PLND, as illustrated in the consort flow diagram (Figure 1). Inclusion criteria for the limited group (2000–2008) included newly diagnosed high‐risk PCa according to D'Amico classification, confirmed by transrectal ultrasound‐guided biopsy with Gleason sums ranging from six to ten, clinical stage T1–T3, PSA up to 100 ng/ml, no evidence of metastatic disease on bone scans prior to PLND, life expectancy greater than 10 yr, and N0 nodal status confirmed by limited PLND of the obturator fossa. According to the Swedish national guidelines in effect at the time of the study, EBRT was not considered for patients in high‐risk settings unless PLND had been performed; therefore, no‐PLND EBRT comparator group was not available for inclusion in the study. Patients who were staged as N0 by a limited PLND prior to receiving EBRT constituted the standard‐of‐care group in this study between 2000 and 2008.

The inclusion criteria for the extended PLND group (2008–2013) were identical to those of the limited group, with the exception that N0 status within the obturator fossa was determined by extended PLND. Only N0 patients, as determined by limited PLND, were eligible for EBRT with curative intent. Notably, patients classified as N0 in the obturator fossa based on findings from an extended PLND prior to EBRT constituted the experimental group from 2008 to 2013.

Nodal status outside the obturator fossa was not a determining factor for inclusions. Consequently, patients in the extended PLND group with histopathologically confirmed lymph node metastases (N1) located outside the obturator fossa were also enrolled.

Exclusion criteria for both included evidence of distant metastatic disease as determined by bone scans or other radiological investigations, lymph node involvement within the obturator fossa, clinical tumour stage T4, age over 75 yr, life expectancy less than 10 yr and contraindication to surgery or EBRT prior to PLND. Cross‐sectional imaging performed prior to PLND was not considered in the patient selection.

Primary clinical data collected included patient age at time of surgery, therapy initiation date, PSA level at diagnosis, clinical tumour stage and the calculated risk for lymph node involvement based on the Briganti nomogram.^17^ Initial treatment parameters, such as the administered dose of EBRT and the duration of androgen deprivation therapy (ADT), were also recorded. Pathological data included Gleason sum score, the number and anatomical location of harvested lymph nodes, and the number of lymph nodes with metastatic involvement.

Patient follow‐up included PSA testing every six months for the first two yr, followed by annual assessments thereafter. BCR was defined according to the Phoenix criteria as a PSA rise of ≥2 ng/ml above nadir, confirmed by two consecutive measurements at least one month apart. In case of PSA progression, radiological cross‐sectional imaging or bone scans were performed and repeated until distant metastases (M1) were confirmed. No radical prostatectomy was offered as a salvage treatment in this study. Adjuvant treatment was administered in accordance with standard care protocols at that time. Outcomes recorded included BCR‐free survival, M1‐free survival, cancer‐specific survival (CSS) and overall survival (OS).

### 2.3 Surgical procedure:PLND

In the limited PLND group, lymph node dissection was performed in the obturator fossa using either an open or laparoscopic approach. In the extended PLND group, lymph nodes were removed according to the anatomical template described by Mattei et al.,^2^ using a laparoscopic approach. Tissue specimens were submitted for pathological examination in separate containers, based on their anatomical location.

### 2.4 Histopathological examination

Lymph nodes were meticulously identified and manually extracted. Nodes with a sagittal diameter less than 10 mm were submitted in the fixating solution in their entirety, while those measuring 10 mm or more were sectioned sagittally before fixation. Fibrotic tissue that could not be reliably distinguished from lymph nodes on macroscopic inspection was also prepared for histological evaluation. Routine staining with haematoxylin and eosin was performed, and immunohistochemistry was used when necessary. The number of lymph nodes retrieved from each anatomical location, as well as the number of metastatic lymph nodes, was recorded.

### 2.5 External beam radiation therapy

Radiation therapy was initiated approximately three months after PLND in both groups. In the limited PLND group, seven participants received a radiation dose below 78 Gy, 25 received 78 Gy and 55 received 80 Gy. Additionally, three participants underwent combination therapy with brachytherapy and EBRT. Extended field radiation, including portions of the obturator fossa, was administered to 19 patients.

In the extended PLND group, 75 participants received a radiation dose of 80 Gy, of whom 10 also received extended field radiation targeting parts of the obturator region. Combination therapy with brachytherapy and EBRT was given to two participants.

### 2.6 Hormonal therapy

Neoadjuvant ADT using gonadotropin‐releasing hormone was initiated in all participants at the conclusion of surgery. Among the early patients in the limited PLND group, a total of 28 did not receive adjuvant ADT. In the limited PLND group overall, the duration of adjuvant ADT ranged from 6 to 18 months. In the extended PLND group, six patients received lifelong hormonal therapy, while in the remaining patients, the duration of ADT ranged from 6 months to 3 years.

Androgen receptor pathway inhibitors (ARPi) were approved in Sweden in 2016 for the treatment of metastatic castration‐resistant PCa. From 2016 onward, 23 events of BCR, M1 or CSM were observed—14 in the limited PLND group and nine in the extended PLND group. ARPi therapy was administered to five patients in the limited PLND group, four of whom subsequently experienced CSM. In the extended PLND group, two patients received ARPi, and both ultimately died from PCa. ARPi, chemotherapy or palliative radiotherapy were used in advanced stages of the disease.

### 2.7 Sample size and statistical analysis

The primary objective was to compare the BCR‐free survival between the limited and extended PLND groups. Secondary objectives included M1‐free survival, CSS and OS. The study was initially designed to detect a 50% improvement in BCR‐free survival, requiring a minimum of 90 patients per group to achieve 80% statistical power. However, an estimated censoring rate of 50% in the limited PLND group was anticipated to impact the effective sample size and statistical power.

Continuous variables were summarized using descriptive statistics, including means with standard deviations (SD) for normally distributed data and medians with interquartile ranges (IQR) for non‐normally distributed data. Categorical variables were described using absolute and relative frequencies. Comparisons of continuous variables between groups were conducted using Student's t‐test for normally distributed data and the Mann–Whitney U test for non‐normally distributed data. Categorical variables were compared using the Chi‐square test.

Survival outcomes (BCR‐free survival, M1‐free survival, CSS and OS) were analysed using Kaplan–Meier survival curves, with differences between groups assessed by the log‐rank test. To determine whether the extent of PLND was independently associated with these endpoints, multivariable Cox proportional hazards regression models were constructed, adjusting for age at surgery (continuous) and Cambridge prognostic group (CPG, categorical). Hazard ratios (HRs) and corresponding 95% confidence intervals (CIs) were reported. Mood's test was used to compare differences in the median duration of adjuvant ADT and in the radiation dose between the two groups.

To evaluate the effect of the extended PLND intervention, relative risk (RR), risk difference (RD) and numbers needed to treat (NNT) were calculated, each with corresponding 95% CIs.

All statistical tests were two‐sided, and a p‐value of <0.05 was considered statistically significant. Analyses were performed using SPSS Statistics version 29 (IBM SPSS, New York, NY, USA) and R statistical software (v4.0.5; R Core Team 2021).^18^

## 3 RESULTS

The analysis included 90 patients in the limited PLND group and 77 in the extended PLND group. Further enrolment in the extended PLND group was discontinued due to a change in clinical practice, which no longer required PLND prior to EBRT. No participant was lost to follow‐up in either group. Patient characteristics are summarized in Table 1. The extended PLND group was older, with a mean age of 66.8 yr compared to 64.2 yr in the limited PLND group (*p =* 0.004). A higher proportion of patients in the extended PLND group had a Gleason sum of seven compared to the limited PLND group (*p =* 0.0032). However, no significant difference was observed in the distribution of CPG four and five between the two groups (*p =* 0.21) (Table 1). The mean number of harvested lymph nodes in the extended PLND group was nearly three times that of the limited PLND group, with averages of 18.8 and 5.8 nodes per patient, respectively. Nodal positivity was confirmed in 35% (*n* = 27) of participants in the extended PLND group, with positive nodes located outside the obturator fossa (Table 1). Rates of nodal positivity were similar between CPG 4 and CPG 5 in the extended PLND group, at 36% and 34%, respectively (Table 1).

**TABLE 1:** Patient characteristics and investigational results.

	Limited PLND	Extended PLND	*p*
Patients, *n*	90	77	
Age (yr), *mean (SD)*	64.2 (6.0)	66.8 (5.6)	0.004^a^
PSA (ng/mL), *median (IQR)*	21.5 (15.75–32.0)	23 (11.0–36.0)	0.90^b^
cT‐stage, *n (%)*			0.08^c^
T1c	15 (16.7)	6 (7.8)	
T2	15 (16.7)	15 (19.5)	
T3	60 (66.7)	56 (72.7)	
Biopsy Gleason Score, *n (%)*			0.0032^c^
6	28 (31.1)	8 (10.4)	
7	43 (47.8)	53 (68.8)	
8–10	19 (21.1)	16 (20.8)	
Briganti risk % for nodal involvement			0.036^b^
*median (IQR)*	56 (41.5)	59.0 (37.5)	
Cambridge Prognostic Group, *n (%)*			0.21^c^
4	48 (53.3)	33 (42.9)	
5	42 (46.7)	44 (57.1)	
Total lymph nodes, n	518	1449	‐
*mean (SD)*	5.8 (2.9)	18.8 (6.5)	
Obturatorius lymph nodes, n	518	572	<.001^a^
*mean (SD)*	5.8 (2.9)	7.4 (3.5)	
Participants with N1, n (%)	‐	27 (35.1)	‐
Participants with N1 in CPG4, n (%)	‐	12 (36.4)	‐
Participants with N1 in CPG5, n (%)	‐	15 (34.1)	‐
Nodal involvement in obturator fossa, n	0	0	‐
Nodal involvement outside obturator fossa, n	Unknown	50	‐
Distribution of N1 per participant, n (%)			‐
1 node	‐	12 (44.4)	
2 nodes	‐	9 (33.3)	
3 nodes	‐	4 (14.8)	
4 nodes	‐	2 (7.4)	

SD: Standard deviation IQR: Interquartile range cT: Clinical Tumour stage CPG: Cambridge prognostic group PLND: Pelvic lymph node dissection N1: lymph node positive disease.

^a^ Student's t‐test.

^b^ Mann‐Whitney U test.

^c^ Chi‐square test.

The median duration of adjuvant ADT in the entire cohort was nine months. Mood's test demonstrated a significant difference in median adjuvant ADT duration between the groups (p < 0.001), with a shorter duration in the limited PLND group compared with the extended PLND group (6 vs. 24 months). Among the six patients in the extended PLND group who received lifelong adjuvant ATD, five were node‐positive. Data on adjuvant hormonal treatment were missing for one patient in the limited PLND group and for six patients in the extended PLND group. In contrast, EBRT dose distribution did not differ significantly between the groups, as most patients received 78–80 Gy (p = 0.43).

### 3.1 BCR rates

**FIGURE 2:**
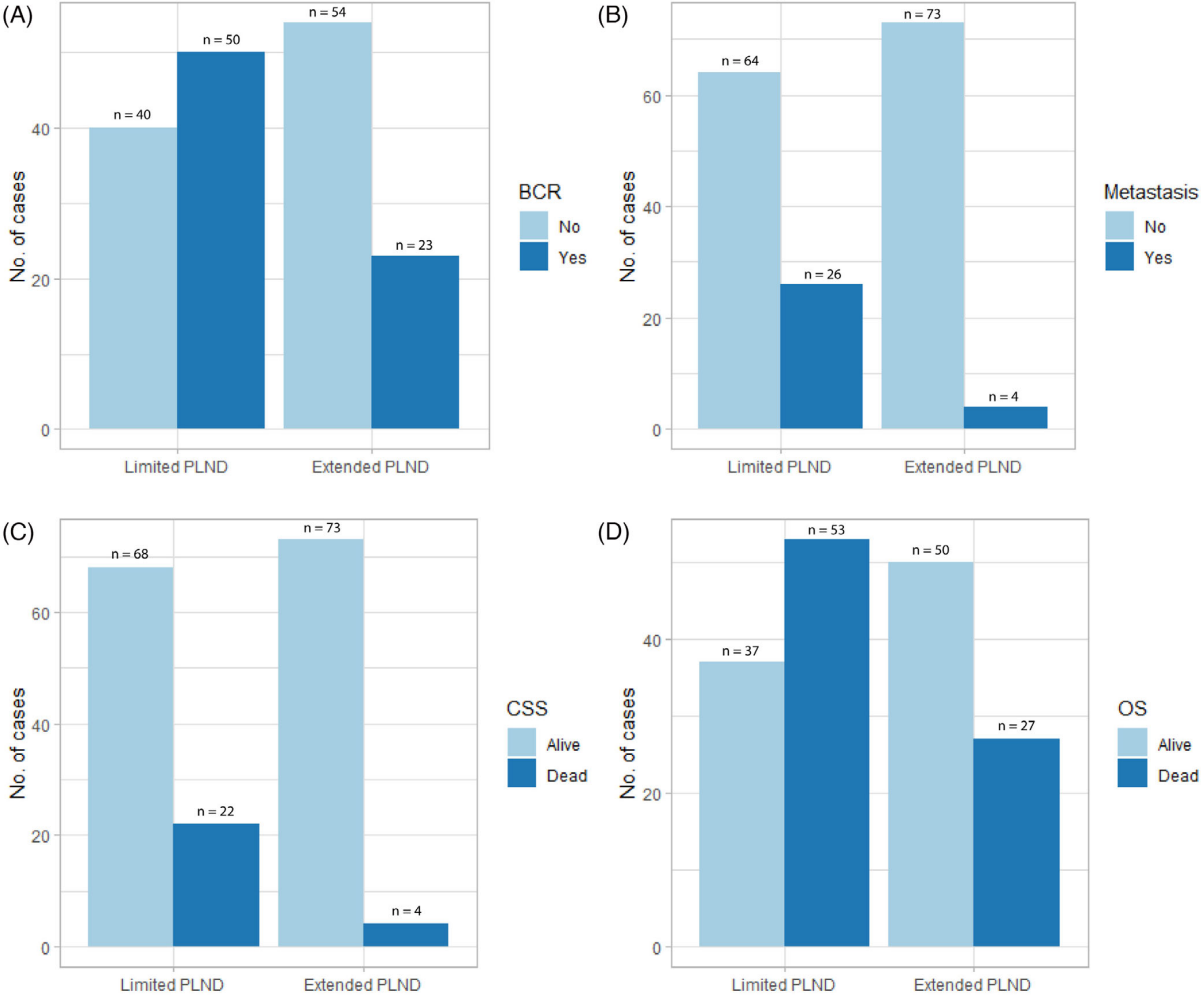
Number of events in the limited and extended group concerning a) biochemical relapse (BCR), b) distant metastases (M1), c) cancer‐specific survival (CSS), and d) overall survival (OS).

**FIGURE 3:**
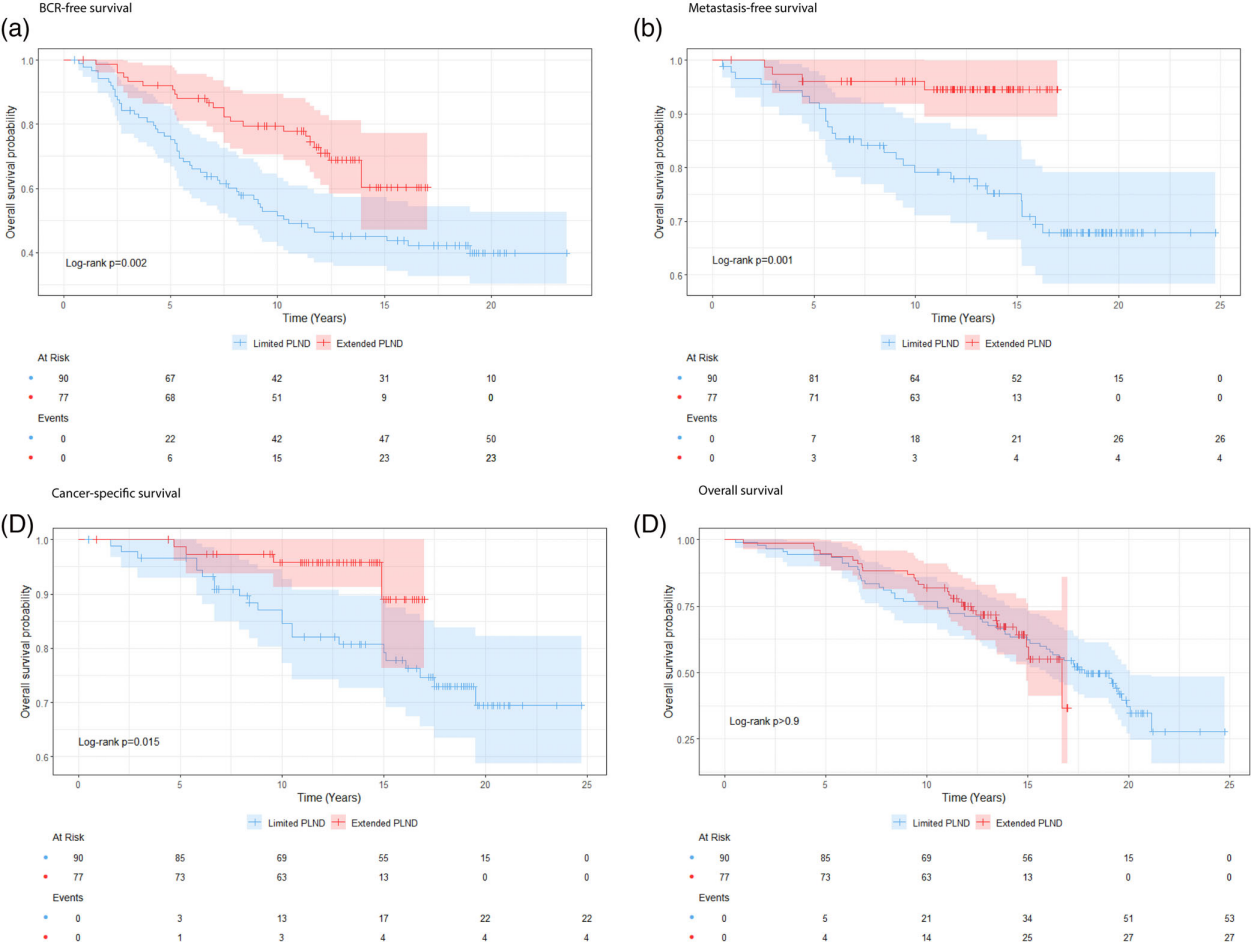
Kaplan‐Meier survival curves illustrating a) biochemical relapse (BCR)‐free survival, B) metastasis‐free survival, C) cancer‐specific survival and D) overall survival, stratified by limited and extended PLND. The survival probability is plotted against time (in yr), with tick marks indicating censored observations. The log‐rank test was used to compare survival distributions between groups. 95% confidence intervals are shown in the plot.

The study demonstrated a significantly lower incidence of BCR in the extended PLND group compared to the limited PLND group (n = 23 vs. n = 50, respectively), Figure 2. At the 11‐year follow‐up, the extended group showed a significantly reduced relative risk of BCR (RR: 0.43, 95% CI: 0.26–0.69) with 44 events observed in the limited PLND group and 16 in the extended PLND group. This corresponds to an absolute risk reduction of 28.1% (95% CI: 42–14) in favour of the extended PLND group (Table 2). Multivariable adjusting Cox regression analysis, adjusting for age at surgery and CPG score, confirmed a significantly lower hazard of BCR in the extended PLND group (HR: 0.51, 95% CI: 0.31–0.86; Table 3). Kaplan–Meier analysis demonstrated significantly improved BCR–free survival in the extended PLND group compared with limited PLND (log‐rank p = 0.002; Figure 3).

**TABLE 2:** Follow‐up data.

	Limited PLND (n = 90)	Extended PLND (n = 77)	Relative risk (95% CI)	NNT (95% CI)	Risk difference (95% CI)	*p*
BCR						
No. of events	50	23				<0.001^a^
Mean follow‐up, yr (SD)	10.6 (6.8)	10.8 (4.1)				0.82^b^
11 yr follow‐up			0.43 (0.26–0.69)	3.57 (2.38–7.08)	−0.28 (−0.42 ‐ ‐0.14)	
No. of events	44	16				<0.001^a^
Cumulative incidence (%)	48.9	20.8				
M1						
No. of events	26	4				<0.001^a^
Mean follow‐up, yr (SD)	14.2 (6.3)	12.1 (3.5)				0.008^b^
11 yr follow‐up			0.26 (0.09–0.73)	6.75 (4.03–21.0)	−0.15 (−0.24 ‐ ‐0.05)	
No. of events	18	4				0.004^a^
Cumulative incidence (%)	20	5.2				
CSM						
No. of events	22	4				<0.001^a^
Mean follow‐up, yr (SD)	14.8 (5.8)	12.3 (3.3)				<0.001^b^
11 yr follow‐up			0.27 (0.08–0.91)	9.48 (5.17–57.57)	−0.11 (−0.19 ‐ ‐0.02)	
No. of events	13	3				0.028^a^
Cumulative incidence (%)	14.4	3.9				
OM						
No. of events	53	27				0.002^a^
Mean follow‐up, yr (SD)	14.9 (5.8)	12.3 (3.3)				<0.001^b^
11 yr follow‐up			0.78 (0.43–1.43)	19.41 (13.87–5.71)	−0.05 (−0.17–0.07)	
No. of events	21	14				0.43^a^
Cumulative incidence (%)	23.3	18.2				

PLND: Pelvic lymph node dissection NNT: Number needed to treat CI: Confidence interval SD: Standard deviation BCR: Biochemical relapse M1: Distant metastases CSM: Cancer specific mortality OΜ: Overall mortality.

^a^ Chi‐square test for proportions.

^b^ Student's t‐test.

**TABLE 3:** Cox regression model results.

	Unadjusted model		Adjusted model^a^	
	HR (95% CI)	*p*	HR (95% CI)	*p*
BCR				
Limited PLND	1 (ref)		1 (ref)	
Extended PLND	0.47 (0.29–0.78)	0.003	0.51 (0.31–0.86)	0.01
M1				
Limited PLND	1 (ref)		1 (ref)	
Extended PLND	0.21 (0.07–0.59)	0.004	0.22 (0.08–0.65)	0.006
PCSM				
Limited PLND	1 (ref)		1 (ref)	
Extended PLND	0.28 (0.09–0.83)	0.022	0.31 (0.10–0.92)	0.035
OS				
Limited PLND	1 (ref)		1 (ref)	
Extended PLND	1.01 (0.61–1.68)	0.9	0.92 (0.55–1.54)	0.7

HR: Hazard ratio CI: Confidence interval BCR: Biochemical relapse M1: Distant metastasis PCSM: Prostate specific cancer mortality OS: Overall Survival PLND: Pelvic lymph node dissection.

^a^ Adjusted for age at surgery (continuous) and Cambridge prognostic group (4 vs 5).

### 3.2 Metastatic disease

The incidence of M1 was significantly lower in the extended PLND group compared to the limited PLND group (n = 4 vs n = 26, respectively), Figure 2. At the 11‐year follow‐up, the relative risk of developing metastatic disease was significantly reduced in the extended PLND group (RR: 0.26, 95% CI: 0.09–0.73) with 18 observed events in the limited PLND group and four in the extended PLND group. This corresponds to an absolute risk reduction of 15.0% (95% CI: 24–5) in favour of the extended PLND group (Table 2). In the multivariable Cox regression analysis, adjusting for age at surgery and CPG score, the extended PLND group showed a lower hazard of developing distant metastases (HR: 0.22, 95% CI: 0.08–0.65) (Table 3). Kaplan–Meier analysis demonstrated significantly improved M1–free survival in the extended PLND group compared with limited PLND (log‐rank p = 0.001; Figure 3).

### 3.3 N1 and survival in the extended PLND group

The distribution of nodal involvement in the extended PLND group is shown in Table 1. Among the 27 participants with histologically confirmed nodal metastases, only three prostate cancer‐specific deaths were observed. In total, four participants in the extended PLND group died of prostate cancer, with none, one, two and four metastatic lymph nodes identified at the time of diagnosis, respectively. The median follow‐up time without cancer‐specific death in the N1 subgroup was 12.3 yr (IQR: 4).

### 3.4 Disease progression

In the limited PLND group, 52% of patients who experienced BCR progressed to metastatic disease (M1), compared to 17% in the extended PLND group. Furthermore, 84% of patients with M1 in the limited PLND group and 100% of those with M1 in the extended PLND group progressed to cancer‐specific mortality (Figure 2).

### 3.5 Cancer‐specific survival rates

Significantly lower CSM was observed in the extended PLND group compared to the limited PLND group. The 11‐year follow‐up analysis demonstrates a lower relative risk for CSM 0.27 (95% CI: 0.08–0.91) with an absolute risk reduction of 11% (95% CI: 19–2) Table 2, with 22 observed events in the limited PLND group and four in the extended PLND group, Figure 2. Consistent with the findings for BCR and distant metastasis, multivariable Cox regression analysis, adjusting for age at surgery and CPG score, demonstrated a significantly lower hazard of prostate CSM in the extended PLND group (HR: 0.31, 95% CI: 0.10–0.92) (Table 3). Kaplan–Meier analysis demonstrated significantly improved CSS in the extended PLND group compared with limited PLND (log‐rank p = 0.015; Figure 3). In the limited PLND group, the number of CSM events among patients who did not receive adjuvant ADT was 13, compared with nine among those who did receive adjuvant ADT.

### 3.6 Overall survival

Regarding OM, 53 events were observed in the limited PLND group and 27 in the extended PLND group (Figure 2). At the 11‐year follow‐up, no statistically significant difference in relative risk was found between the groups (RR: 0.78, 95% CI: 0.43–1.43). The absolute risk reduction was 5% (95% CI: 17–7), which was not statistically significant and included potential benefit to harm (Table 2). Similarly, the multivariable Cox regression analysis adjusted for age at surgery and CPG score revealed no significant difference in the hazard of overall survival between the extended and limited PLND groups (HR: 0.92, 95% CI: 0.55–1.54) (Table 3). Kaplan–Meier analysis also demonstrated no significantly improved OS in the extended PLND group compared with limited PLND (log‐rank p > 0.9; Figure 3). Cardiovascular mortality accounted for 22% of overall mortality in the limited PLND group compared with 29% in the extended PLND group; however, this difference was not statistically significant (p = 0.49). In contrast, mortality due to other cancers represented 11.3% of overall mortality in the limited PLND group and 37% in the extended PLND group, a statistically significant difference (p = 0.006).

### 3.7 Discussion

This study suggests that extended PLND prior to curative‐intent EBRT in patients with high‐risk PCa may be beneficial, as evidenced by significantly lower rates of BCR, metastatic progression and cancer‐specific mortality compared to limited PLND. However, no significant difference in OS was observed between the groups. The substantial reduction in metastatic disease progression and cancer‐specific deaths supports the notion that extended PLND may enhance long‐term disease control, particularly in the 10–15 year period following definitive treatment (Figure 3). The observed relative and absolute risk reductions further reinforce the hypothesis that removal of metastatic lymph nodes contributes to improved oncologic outcomes and, in selected cases, may have a curative effect. However, when interpreting these results, it is important to note that the cohort primarily consisted of patients with low‐volume nodal disease.

Our findings are consistent with previous observational studies that have reported survival benefits associated with PLND performed at the time of radical prostatectomy.^19^, ^20^, ^21^, ^22^ Retrospective data suggest that patients with low‐volume nodal metastases, typically defined as two or three lymph nodes, may still benefit from curative‐intent treatments, while those with high‐volume nodal disease do not appear to experience a survival advantage from such approaches.^23^, ^24^, ^25^ Additionally, other studies have indicated that early initiation of ADT following primary curative therapies in patients with nodal involvement (N1) is associated with both improved survival and reduced risk of relapse.^26^ ^,^ ^27^

In a randomized trial by Lestingi et al. (2020), improved BCR outcomes were observed only in a subgroup of patients with ISUP grade 3–5 who were allocated to the extended PLND arm.^8^ No other survival benefit was demonstrated, likely due to the short follow‐up period and the low number of N1 participants, only 25 (17%) out of 150 in the extended PLND arm and 5 (3.4%) out of 150 in the limited arm. Notably, our results are partially consistent with the 2024 update of the Touijer trial, which reported improved metastasis‐free survival in the extended PLND arm after a median follow‐up of 5.4 yr.^10^ However, in that trial, the rate of lymph node involvement, the median number of lymph nodes removed and the number of participants with nodal metastases did not differ significantly between the groups. Furthermore, the overall proportion of participants with lymph node involvement was relatively low in both arms, 12% (81/700) in the limited group and 14% (100/740) in the extended group. These findings underscore the need for improved patient selection criteria in future studies. Our study's longer follow‐up period and specific focus on high‐risk patients may explain the differences in the observed outcomes. Our findings support the use of extended PLND prior to curative EBRT as a potential strategy to improve oncological outcomes in patients with high‐risk PCa and limited metastatic burden.

### 3.8 Strengths and limitations

To our knowledge, this is the first long‐term study comparing extended and limited PLND performed prior to curative EBRT in patients with high‐risk PCa. Importantly, since local treatment in this cohort was based on radiotherapy rather than surgery, the outcomes are not influenced by positive surgical margins, a possible confounding factor in studies involving radical prostatectomy, where salvage radiotherapy and ADT may be required.^15^ Additionally, the high number of lymph nodes harvested per patient, combined with a relatively high incidence of N1 in the extended PLND group, provides a robust histological foundation for evaluating nodal disease and treatment outcomes.

This study is not without limitations. First, the retrospective nature of cohort assembly, despite prospective follow‐up, may introduce temporal and selection bias. Although both groups were carefully matched for nodal negativity in the obturator fossa, the differing inclusion periods may reflect evolving clinical practices over time. However, the most pronounced difference between the groups was observed in BCR. ADT was reinitiated at the clinicians' discretion throughout the study period, in accordance with the standard clinical practice at the time. No additional systemic treatments after recurrence were considered during the course of the study.

Variations in the EBRT dosage between groups represent another potential source of bias, particularly in the early patients in the limited group. These variations are attributable to the introduction of intensity‐modulated and image‐guided radiotherapy, which allowed for the safe delivery of higher doses. However, only a small number of patients (n = 7) received a radiation dosage lower than 78–80 Gy, and this difference was not statistically significant. Additionally, patients in the extended group received slightly longer durations of adjuvant hormonal therapy, as the recommended duration of adjuvant ADT was progressively extended over the study period. The long‐term impact of this difference is difficult to interpret within the context of this study. However, this represents a potential source of bias, as evidence suggests a survival advantage with three years of ADT compared with six months^28^ in clinically node‐negative settings. In contrast, randomized data from Granfors et al. (2006) demonstrated no difference in mortality among node‐negative patients who underwent limited PLND and received radiation therapy plus orchiectomy compared with those treated with radiation therapy followed by deferred ADT at the time of disease progression.^12^

Baseline differences in age and Gleason sum between the groups may also pose a confounding risk, potentially biasing survival outcomes against the extended group. However, these variables were accounted for in multivariable Cox regression analyses. Specifically, the imbalance in the Gleason sum of six cases may be partly explained by changes in the histological grading criteria for prostate cancer over time.^29^ Moreover, the groups were comparable according to the Cambridge Prostate Group stratification system. Specifically, the absence of differences in OS rates may have been influenced by the older age of the extended PLND cohort and the higher mortality attributable to other malignancies.

Data on treatment‐related complications, such as lymphedema, were sparsely documented and therefore not reported. Patient inclusion in the extended group was prematurely halted due to changes in clinical guidelines for the management of high‐risk PCa.^16^ Finally, while the sample size was adequately powered to detect differences in BCR, it may be underpowered to detect statistically significant differences in other endpoints, such as metastasis‐free or overall survival.

In conclusion, the present study shows reductions in biochemical recurrence, metastatic progression and cancer‐specific mortality long‐term outcomes following limited versus extended pelvic lymph node dissection in patients with high‐risk prostate cancer and N0 status in the obturator fossa, prior to curatively intended external beam radiation therapy. Future randomized controlled trials with long‐term follow‐up and standardized treatment protocols are essential to validate these results and to more clearly delineate the role of extended PLND within the multimodal treatment paradigm for high‐risk PCa.

## AUTHOR CONTRIBUTIONS

**Georgios Daouacher:** Supervision; data curation; formal analysis; validation; writing—original draft; writing—review and editing. **Jessica Carlsson:** Formal analysis; writing—review and editing. **Pernilla Sundqvist:** Supervision; writing—review and editing. **Mauritz Waldén:** Conceptualization; methodology; data curation; investigation; supervision; writing—review and editing.

## DECLARATION OF INTERESTS

All authors have completed the ICMJE uniform disclosure form, and declare no support from any organization for the submitted work; no financial relationships with any organizations that might have an interest in the submitted work; and no other relationships or activities that could appear to have influenced the submitted work.
